# Shunting of Oxygenated Blood to the Venous System in the Avalon® Cannula on Venovenous Extracorporeal Membrane Oxygenation with High-frequency Oscillatory Ventilation

**DOI:** 10.7759/cureus.3661

**Published:** 2018-11-30

**Authors:** Lydia McDermott, Nicholas C Cavarocchi, Hitoshi Hirose

**Affiliations:** 1 Surgery, Thomas Jefferson University, Philadelphia, USA; 2 Cardiothoracic Surgery, Thomas Jefferson University, Philadelphia, USA

**Keywords:** high-frequency oscillatory ventilation (hfov), acute respiratory distress syndrome (ards), extracorporeal membrane oxygenation (ecmo), avalon cannula

## Abstract

High-frequency oscillatory ventilation (HFOV) may assist in the prevention of volutrauma for high-risk patients with acute respiratory distress syndrome (ARDS) during venovenous extracorporeal membrane oxygenation (VV ECMO). In combined VV ECMO and HFOV, we noted that increased intrathoracic pressure contributed to shunt formation in the dual-lumen Avalon® cannula (Maquet, Rastatt, Germany).

A 51-year-old female with ARDS secondary to aspiration pneumonia was placed on VV ECMO using a single Avalon cannula. By ECMO Day 16, she became unable to ventilate due to elevated peak airway pressures, even with low tidal volume ventilation and an otherwise stable VV ECMO course. HFOV was introduced to minimize ventilator-induced lung injury. Shortly after HFOV started, the patient desaturated, and consequently, the fraction of inspired oxygen (FiO_2_) was increased to 100%. We noted that a flash of bright red, oxygenated blood was flowing retrograde in the Avalon cannula at the same rate as the beat of the oscillator, while the patient's ECMO flow rate, arterial blood gas, and blood pressure all remained stable. The ECMO flow was increased above 5.5 L/min and the resolution of the retrograde shunt through the Avalon cannula was immediately observed.

Concurrent use of HFOV with VV ECMO using an Avalon cannula may result in a shunt that becomes visible with arterial O_2_ saturations nearing 100%. Due to pressure differences between the venous and arterial lumens of the Avalon cannula, increasing the ECMO flow rate appeared to decrease this shunting effect caused by elevated intrathoracic pressure.

## Introduction

In addition to removal of the initial insult, treatment of acute respiratory distress syndrome (ARDS) consists primarily of symptom management. Conventionally, lung protective ventilation practices are used to decrease further volutrauma, while extracorporeal membrane oxygenation (ECMO) is introduced for added support in cases complicated by refractory hypoxia and/or hypercarbia. Common additional adjuncts include prone therapy, deep sedation and paralysis, and nebulized vasodilators.  

For ARDS patients with high airway pressures intractable to maximum therapies, including lung protective ventilation and ECMO, high-frequency oscillatory ventilation (HFOV) should be considered [1-3]. HFOV provides a small tidal volume (1 - 4 ml/kg) in high-frequency (3 - 15 Hz) to optimize alveolar gas exchange while eliminating peak pressure and full pulmonary expansion in the setting of restricted compliance.  

Our current venovenous extracorporeal membrane oxygenation (VV ECMO) practice includes the use of a single, dual-lumen Avalon® cannula (Maquet, Rastatt, Germany) placed to the right internal jugular vein, although with this positioning, the applied intrathoracic pressure created by HFOV may compete with the ECMO flow. Herein, we report a case of blood shunting observed in an Avalon cannula and treated with an increase of ECMO flow.

## Case presentation

A 51-year-old female (weight: 73.5 kg; height: 160 cm) with ARDS secondary to aspiration pneumonia was placed on VV ECMO using a single 27 Fr Avalon cannula to the right internal jugular vein. Her peak airway pressure was 46 cm H_2_O, even with low tidal volume (200 ml) ventilation, and eventually, she was unable to ventilate safely due to decompensated compliance. HFOV with a frequency of 300 bpm and 5 Hz was introduced on ECMO Day #16 to decrease the risk of volutrauma while also preventing atelectasis from hypoventilation. Her mean airway pressure (mPaw) became 29.3 cm H_2_O with HFOV, which comparatively had been 16 cm H_2_O on the conventional ventilator. At the time of transition to HFOV, her settings were: ECMO flow 4.56 L/min, Sweep 6 L/min, FiO_2_ 70%, with ventilator FiO_2_ 50%. Approximately two hours later, the patient desaturated requiring FiO_2_ 100% on both the ECMO and HFOV to maintain an O_2_ saturation (SaO_2_) of 85%, although the ECMO flow was maintained at 4.5 L/min. These same settings were continued before a flash of bright red, oxygenated blood was noted flowing into the venous return lumen of the Avalon cannula which synchronized with each beat of the oscillator (Video 1). 

**Video 1:** Avalon cannula Flash of bright red, oxygenated blood is noted flowing into the outflow lumen in the patient from the Avalon extracorporeal membrane oxygenation (ECMO) cannula (yellow arrow), suggesting recirculation of oxygenated and unoxygenated blood.

The correct placement of the Avalon catheter and endotracheal tube were confirmed by chest x-ray, and an echocardiogram further confirmed the cannula position (the tip in the inferior vena cava and the access lumen facing the tricuspid valve), as well as ruled out a patent foramen ovale or an atrial septal defect. Inter-atrial shunting within the Avalon cannula was diagnosed, and the ECMO flow was increased above 5.5 L/min to overcome the additional resistance. This provided a resolution of the retrograde shunt. Despite all efforts, this patient, unfortunately, expired due to multi-organ failure. Her family elected to withdraw care after a total of 20 days on ECMO and four days on HFOV.

## Discussion

It was hypothesized that the pulses of applied intrathoracic pressure created by the HFOV contrasted with the systemic low pressures of the venous system where the Avalon cannula lies, causing the intra-atrial shunt. As described by Sklar et al., elevation of the right atrial (RA) and pulmonary artery pressures (PAP) were observed during HFOV [4]. In an ECMO circuit, negative pressure (approximately -80 mmHg assuming an ECMO flow of 4.5 L/min with an Avalon cannula size 27 Fr) was maintained through the return lumen while positive pressure (approximately 240 mmHg assuming an ECMO flow of 4.5 L/min with an Avalon cannula size 27 Fr) is applied by the centrifugal pump into the access lumen. Using the Avalon cannula allows the circuit to suction de-oxygenated blood from the superior vena cava (SVC) and inferior vena cava (IVC) and instill oxygenated blood toward the tricuspid valve simultaneously creating a VV ECMO system through only one insertion site. With augmented external pressure from HFOV, the PAP suddenly elevates and causes the Avalon catheter’s oxygenated out-flows to face an increased resistance which can dilate the RA when sustained. Thus, turbulent flow within the RA may cause shunting that can exacerbate hypoxia as oxygenated blood recirculates towards the venous return lumen of the Avalon cannula. In our patient, we believe the intra-atrial recirculation became noticeable due to a deepened contrast between the venous and arterial blood as the FiO_2_ from ECMO was raised to 100%.

To decrease PAP, deep sedation and paralysis may be necessary, though these were already being applied in this case. Furthermore, inhaled vasodilators, such as nitric oxide or epoprostenol, are an additional adjunct; however, we have found this therapy to be impractical during HFOV use. Instead, we increased the VV ECMO flow above 5.5 L/min, which was able to unload the RA by overcoming the raised PAP (access lumen approximately 400 mmHg) and optimizing suction from the SVC and IVC (return lumen approximately -110 mmHg), resolving the intra-atrial shunt. While maintaining a high ECMO flow is possible, this requires additional patient monitoring and care due to a concomitant heightened risk of mechanical hemolysis. For this reason, we determined that, while interesting to note, intra-atrial shunt presence is clinically relevant to the patient primarily when contributing to decompensated hypoxia refractory to typical management strategies (namely, increasing FiO_2_). Therefore, we elected to continue this patient’s ECMO management per usual protocols by setting the ECMO flow based on the body surface area (BSA) and symptom severity (with the goal to maintain the SaO_2_ above 85% and a normal pH) rather than on the mere presence or visibility of a shunt.  

Upon review of this case, we are reminded that the mPaw on HFOV is higher than that of lung protective ventilation via a conventional ventilator. While this comparative increase is expected due to the need for accommodation of the loss of dynamic PEEP, perhaps the mPaw could have been decreased in a setting of resolved hypoxia. As suggested in Sklar et al., elevated mPaw itself may contribute to volutrauma, potentially explaining the failure of HFOV to be proven beneficial thus far [4]. It should be considered that while lung protective ventilation has been shown to improve ARDS outcomes during conventional ventilation, standard practice settings are applied during HFOV to maintain an adequate gas exchange. Facilitation of lung protective ventilation while on HFOV could be possible with the concurrent use of HFOV and high-flow ECMO, however. While we were unable to attempt this method for our patient due to sustained hypoxia, it may simply be that stricter parameters and a decreased mPaw are required to create a mortality benefit, as well as limit intra-atrial shunting, as we described. Further research is required to evaluate the outcomes of severe ARDS patients on minimized, lung protective HFOV settings. 

It is known that HFOV may promote hemodynamic instability and other sequelae, though once identified, mitigation of these problems may be possible. Regardless, present information suggests that HFOV worsens ARDS mortality for mild-moderate cases and remains unproven as a benefit for severe cases. Its use continues to be up for debate; therefore, it is not recommended as an initial therapy. However, HFOV can be a useful adjunct in patients who continue to fail after conventional ventilator management is maximized with concurrent use of ECMO, paralysis, prone therapy, and nebulized epoprostenol. The expansion of care modalities is critical to continue advancing the ability for high-risk patients to recover from severe episodes of ARDS.

## Conclusions

By maintaining safer pulmonary pressures with HFOV, while improving the exchange of gasses via ECMO, lungs impaired by ARDS that have failed conventional ventilation may be further allowed to heal. In the event that a clinically relevant intra-atrial shunt occurs during the concurrent use of HFOV with VV ECMO via an Avalon cannula, it is likely that an increase in the ECMO flow rate will decrease the shunting effect.
